# AI in Proton Therapy Treatment Planning: A Narrative Review

**DOI:** 10.1016/j.ijpt.2026.101965

**Published:** 2026-09-25

**Authors:** Yuzhen Ding, Hongying Feng, Martin Bues, Mirek Fatyga, Tianming Liu, Wei Zhang, Thomas J. Whitaker, Haibo Lin, Nancy Y. Lee, Charles B. Simone, Samir H. Patel, Daniel J. Ma, Steven J. Frank, Sujay A. Vora, Jonathan B. Ashman, Wei Liu

**Affiliations:** 1 Department of Radiation Oncology, Mayo Clinic, Phoenix, AZ 85054, USA; 2 School of Computing, The University of Georgia, Athens, GA 30602, USA; 3 Department of Computer & Cyber Sciences, Augusta University, Augusta, GA 30912, USA; 4 Department of Radiation Physics, The University of Texas MD Anderson Cancer Center, Houston, TX 77054, USA; 5 New York Proton Center, New York City, NY 10035, USA; 6 Department of Radiation Oncology, Memorial Sloan Kettering Cancer Center, New York City, NY 10065, USA; 7 Department of Radiation Oncology, Mayo Clinic, Rochester, MN 55905, USA; 8 Division of Radiation Oncology, The University of Texas MD Anderson Cancer Center, Houston, TX 77054, USA

**Keywords:** Artificial intelligence, large language model, proton therapy, adaptive radiation therapy

## Abstract

**Purpose:**

Proton therapy provides superior dose conformity compared to photon therapy, but its treatment planning is challenged by sensitivity to anatomical changes, setup/range uncertainties, and computational complexity. This narrative review evaluates the role of artificial intelligence (AI) in improving proton therapy treatment planning, with emphasis on proton-specific requirements for dose-calculation accuracy, robustness, and adaptive decision-making.

**Materials and methods:**

Recent studies on AI applications across the proton therapy planning workflow, including imaging, registration, segmentation, contour assessment, dose prediction/denoising, optimization, adaptive therapy, and large language model (LLM) and multimodal AI were reviewed and summarized by application domain and validation strategy. Both proton-therapy-specific studies and selected general radiotherapy AI studies were included when they introduced methods relevant to proton treatment planning, with attention to the additional validation needed for proton therapy.

**Results:**

AI has shown promise in enhancing imaging for dose calculation, automating contouring and contour assessment, accelerating dose calculation, predicting and denoising dose distributions, supporting robust optimization, and improving adaptive planning workflows. Compared with photon therapy, AI tools in proton therapy require more stringent evaluation because errors in AI-generated images, deformation fields, contours, dose estimates, or optimization outputs may propagate into water-equivalent path length, range uncertainty, scenario-specific dose distributions, and adaptive replanning decisions. Emerging applications, including LLMs and multimodal foundation models, may further support clinical-context integration, treatment-planning documentation, plan review, and workflow coordination. Key limitations include data scarcity, limited generalizability, physical interpretability, uncertainty management, lack of standardized proton-specific benchmarks, and limited prospective validation.

**Conclusion:**

AI is emerging as a key enabler of efficient, consistent, and patient-specific proton therapy treatment planning. Clinical translation will require interpretable and physics-aware models, multi-institutional validation, workflow-level testing, and evaluation using proton-specific endpoints such as HU/RSP accuracy, range error, dose-calculation accuracy, robustness, deliverability, and adaptive decision consistency.

## Introduction

Radiation therapy plays a pivotal role in cancer treatment, offering both curative and palliative benefits by precisely targeting tumors and minimizing unnecessary irradiation to healthy tissues. Among the available modalities, proton therapy has become increasingly attractive for certain cancer disease sites due to its distinct physical characteristics, particularly the Bragg peak and finite range, which allow for highly conformal dose distributions with much smaller exit dose compared to conventional photon therapy. Clinical studies and reviews have reported potential dosimetric or clinical advantages of proton therapy in settings where normal-tissue sparing is especially important, including pediatric malignancies, re-irradiation, and tumors adjacent to critical organs.^1^, ^2^, ^3^, ^4^, ^5^, ^6^, ^7^, ^8^, ^9^ In such cases, minimizing unnecessary dose can potentially reduce long-term toxicities and improve quality of life.

Despite these benefits, proton beams’ sensitivity to tissue heterogeneities, anatomical variations, and setup uncertainties also pose significant challenges for robust treatment delivery. Unlike photon therapy, where dose deposition is less sensitive to small anatomical variations, proton therapy is strongly affected by uncertainties in Hounsfield unit (HU)-to-relative stopping power (RSP) conversion, resulting in beam range uncertainty, and anatomical variations caused by setup uncertainty, intrafractional organ motion, and inter-fraction anatomical changes.^9^, ^10^, ^11^, ^12^, ^13^, ^14^, ^15^, ^16^, ^17^, ^18^, ^19^ Even small changes in patient anatomy or positioning can lead to substantial deviations between the planned and delivered dose distributions, potentially eroding the clinical benefit of proton therapy.^6^, ^8^, ^20^, ^21^, ^22^, ^23^, ^24^, ^25^, ^26^, ^27^, ^28^, ^29^, ^30^, ^31^ Robust optimization strategies have been developed to mitigate these uncertainties, but they often require significant computational time and specialized planning expertise and expert review. Furthermore, the integration of multimodal imaging, contouring, dose calculation, plan optimization, and adaptive decision-making into the proton therapy treatment planning process creates additional challenges in efficiency and standardization, which can potentially hinder timely clinical decision-making. Therefore, methods that can improve image quality, preserve dosimetric reliability, accelerate dose calculation and optimization, and reduce manual workload are particularly relevant for proton therapy.

In recent years, artificial intelligence (AI) and deep learning (DL) have emerged as promising methods to address these challenges in radiation therapy treatment planning. AI-driven approaches have demonstrated the ability to automate critical components of the radiation therapy treatment planning workflow, including image segmentation^32^, ^33^, ^34^/registration,^35^, ^36^, ^37^ dose prediction,^38^, ^39^, ^40^ and plan quality evaluation.^41^, ^42^ In addition, AI-based image reconstruction and image-to-image translation methods have been explored to generate synthetic computed tomography (CT) images from cone-beam CT (CBCT),^43^, ^44^, ^45^ limited-angle CT,^46^, ^47^, ^48^ orthogonal kilovoltage (kV) images,^49^ magnetic resonance imaging (MRI),^50^, ^51^, ^52^ and other complementary imaging modalities.^53^ Many of these AI methods were first developed in general radiotherapy or photon therapy settings. Therefore, their translation to proton therapy requires additional consideration of proton-specific features, like the much higher vulnerability to uncertainties in RSP accuracy and the consequential proton range and anatomical variations, and technical distinction in plan preparation and delivery. Because Monte Carlo (MC) methods are preferred for accurate proton dose calculation, DL methods have been developed to accelerate MC-based/MC-equivalent dose calculations,^54^, ^55^, ^56^ support robust optimization^57^, ^58^ and robustness evaluation,^59^, ^60^ and provide personalized dose distribution predictions.^61^, ^62^ By reducing manual workload and computational burden, AI has the potential to streamline treatment planning, improve consistency across clinics, and enable more adaptive and patient-specific proton therapy.

Several recent reviews have summarized AI applications in radiation oncology and proton therapy, including treatment planning, image guidance, adaptive workflows, and clinical translation.^63^, ^64^, ^65^ Building on these prior efforts, the present review focuses specifically on the proton therapy treatment-planning chain and emphasizes how AI methods interact with proton-specific requirements, including CT-to-stopping-power conversion, range uncertainty, dose-calculation accuracy, robust optimization, and adaptive replanning. We also discuss selected emerging topics, including 2 dimensional (2D)-to-3 dimensional (3D) CT reconstruction, AI-based dose denoising, robustness-aware AI tools, large language model (LLM)/multimodal AI applications, and other proton-specific research directions.

This article is intended as a narrative review rather than a systematic review. The goal is to summarize representative AI methods relevant to the proton therapy treatment-planning workflow and to highlight proton-specific considerations that affect clinical translation. Therefore, we include both proton-therapy-specific studies and selected general radiotherapy AI studies when they introduce methodological concepts directly relevant to proton treatment planning, such as deformable image registration (DIR), auto-segmentation, dose prediction, dose denoising, and AI-based quality assessment (QA). When evidence is derived primarily from photon radiotherapy or general radiotherapy settings, we discuss the additional validation needed before these methods can be transferred to proton therapy. This distinction is important because many AI tasks, such as image segmentation, DIR, and dose prediction, are not unique to proton therapy, but their downstream clinical consequences may be more stringent in proton therapy because small errors can propagate into range estimation, scenario-specific dose calculation, and adaptive replanning decisions.

The manuscript is organized according to the major components of the proton treatment-planning chain: CT preparation, DIR, contouring and AI-based contour QA, dose prediction and dose denoising, optimization, adaptive radiation therapy (ART), LLM/multimodal AI applications, and broader clinical translation challenges. Patient selection, outcome modeling, and broader clinical decision-support systems are important AI applications in proton therapy, but they are not the primary focus of this treatment-planning-focused review. We also discuss current methodological limitations, regulatory considerations, and emerging research directions, with the goal of clarifying both the opportunities and the remaining barriers for clinically reliable AI-assisted proton therapy treatment planning. Figure 1 provides an overview of the AI application areas covered in this review and highlights the proton-specific validation requirements that should be considered across the treatment-planning workflow.

**Figure 1:**
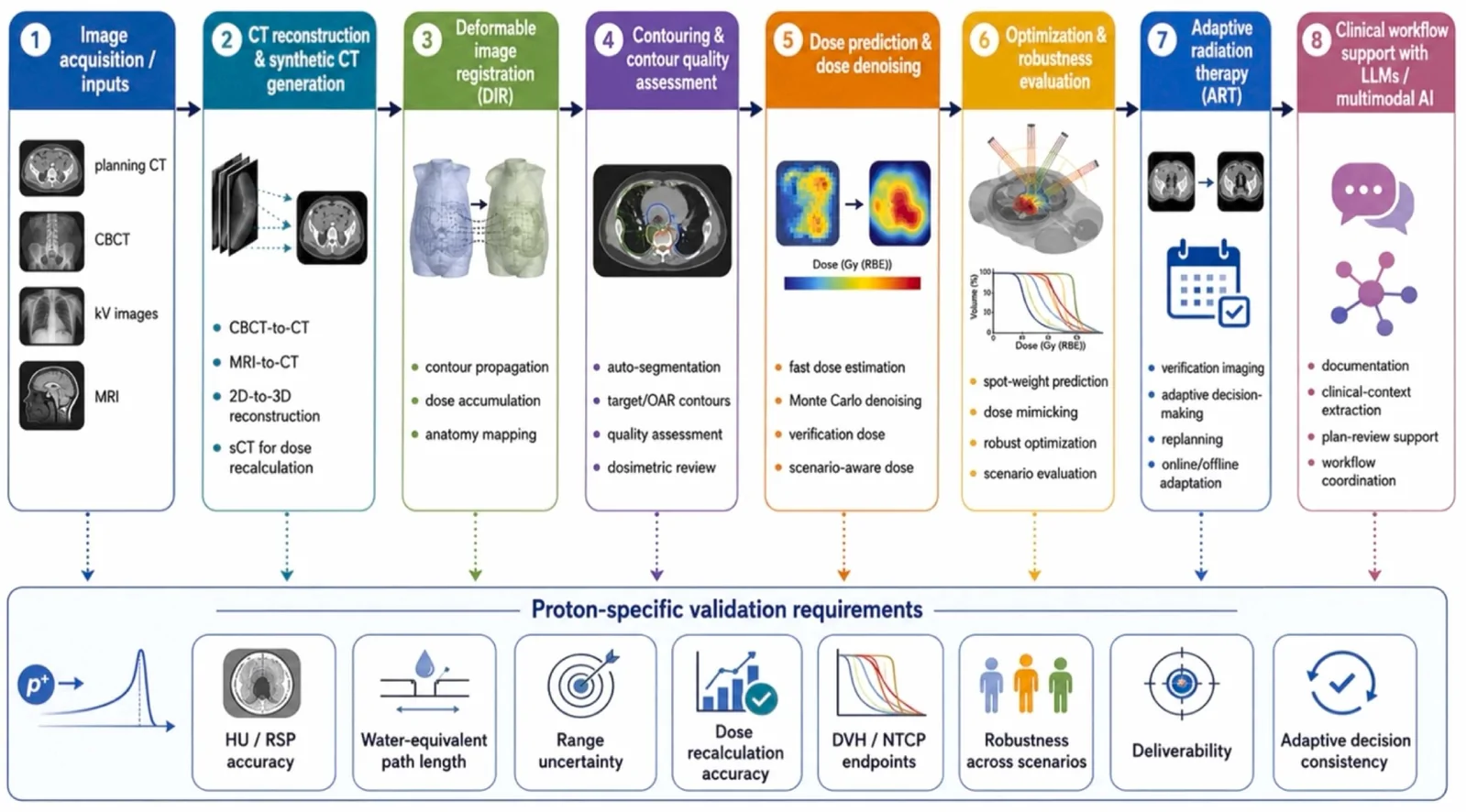
Overview of AI applications across the proton therapy treatment-planning workflow. The figure summarizes key AI application areas and proton-specific validation requirements.

## CT preparation

CT imaging plays a central role in proton therapy treatment planning and delivery. As the primary imaging modality in proton therapy, CT provides detailed anatomical information essential for accurate delineation of tumors and surrounding organs at risk (OARs). In addition to anatomical guidance, CT offers HU values, which can be converted into RSP maps. These RSP maps are critical for calculating the proton beam range and dose distributions. This requirement makes CT image quality and HU accuracy particularly important in proton therapy compared with photon therapy, because proton dose calculation depends directly on the conversion from HU to RSP and on the resulting water-equivalent path length along each beam path.

During the treatment course, repeated on-board imaging (OBI) is commonly used to verify patient positioning and monitor anatomical changes over time. This enables the detection of setup deviations or inter-fractional anatomical variations, which may trigger evaluation for ART. Though indispensable in proton therapy treatment planning, CT has inherent drawbacks that limit its frequent use in clinical practice: long acquisition times and non-negligible radiation dose when used repeatedly. In current radiotherapy practice, several OBI modalities are routinely used, including CT-on-rails (CToR), CBCT, and kV x-ray imaging. CToR provides CT of diagnostic quality but requires transferring the patient from the CT scanner to the treatment isocenter, potentially introducing additional positioning and time error. In contrast, CBCT allows imaging at the treatment isocenter, avoiding patient transfer, but it is more susceptible to scatter, artifacts, and HU inaccuracy that can degrade its direct use for proton dose calculation. Orthogonal kV imaging offers real-time 2D projection images at significantly lower radiation doses and is often used for daily patient setup. However, it provides projected anatomy with limited soft-tissue information, restricting its direct use in treatment planning.

To overcome the limitations of current OBI approaches, AI-based solutions are rapidly emerging and have demonstrated strong potential in enhancing image quality (eg, denoising, artifact correction). These DL models are capable of generating diagnostic-equivalent CT images from lower-dose or lower-quality inputs—such as CBCT, limited-angle CT, or even orthogonal kV x-ray projections—as well as from non-ionizing imaging modalities like MRI and ultrasound. Of note, generating CT from another volumetric image modality is usually referred to as CT synthesizing or image conversion/translation, while generating CT from projected planar images is usually described as 2D-to-3D CT reconstruction. In this review, we therefore refer to them as CT synthesizing and CT reconstruction, with the corresponding resultant image as synthetic CT (sCT) and reconstructed CT (reCT), respectively.

The clinical motivation is not only to improve image guidance but also to make frequently acquired OBI modalities usable, when appropriately validated, for dose recalculation, adaptive decision-making, and potentially adaptive treatment planning. For proton therapy, however, validation must go beyond visual image quality because HU/RSP inaccuracies can directly affect water-equivalent path length, proton range, and dose calculation. Therefore, the key question for AI-generated CT in proton therapy is not simply whether the image possesses diagnostically equivalent geometry quality, but whether it can support clinical decisions such as dose recalculation, range evaluation, robustness assessment, and replanning.

### Synthetic CT generation from 3D inputs

In radiotherapy, early studies of applying AI to sCT generation from 3D inputs often focused on converting CBCT to sCT, as CBCT is routinely acquired for image guidance and shares similar geometric characteristics with diagnostic CT. CBCT-to-sCT translation therefore became a practical starting point for validating the feasibility of DL-based CT synthesizing. Clinically, this task is valuable because a daily or periodic CBCT contains updated information about weight loss, tumor shrinkage, filling changes, and other anatomical variations that may affect proton range and dose distribution. However, raw CBCT is often unsuitable for direct proton dose calculation because scatter, truncation, artifacts, and inaccurate HU values can introduce RSP and range errors. AI-based CBCT-to-sCT generation is therefore intended to combine the anatomical timeliness of CBCT with the HU/RSP reliability needed for dose calculation.

In proton therapy, however, the goal of CBCT-to-sCT or synthetic CT generation is not only to improve visual image quality but also to generate images that are sufficiently accurate for dose calculation. Because CT HU values are converted to RSP, residual HU bias, scatter artifacts, or anatomical mismatch in AI-generated CT images may propagate into RSP errors, proton range errors, and dose-calculation uncertainty. Therefore, evaluation should include image-similarity metrics, such as mean absolute error, structural similarity index measure, or peak signal-to-noise ratio, together with selected proton-relevant endpoints such as HU/RSP accuracy, range error, dose-recalculation accuracy, and adaptive decision consistency. This distinction is less critical for many photon image-guidance tasks, where the image may primarily support positioning or contour assessment, but it is central in proton therapy because inaccurate HU/RSP values may directly shift the Bragg peak or alter distal dose falloff.

In terms of the model utilized, such approaches can be categorized into two general classes, that is, UNet-based and generative adversarial network (GAN)-based. UNet is a convolutional neural network (CNN) architecture with an encoder-decoder structure. A CNN is a neural network architecture that uses convolutional filters to extract spatial image features, making it well suited for medical image analysis tasks such as organ and tumor segmentation. The encoder extracts image features at multiple spatial scales, while the decoder reconstructs the output image. Skip connections transfer spatial information from the encoder to the decoder, helping preserve anatomical detail during image translation. In CBCT-to-sCT synthesis, the network learns to map CBCT image intensities and anatomy to CT-like images. GAN-based methods use a generator to synthesize CT-like images and a discriminator to distinguish generated images from real CT images; through this adversarial training process, the generator is encouraged to produce images with more realistic texture and intensity distributions. CycleGAN is particularly useful when paired CBCT and CT images are unavailable, because it can learn image translation using unpaired data. However, realistic texture alone is not sufficient for proton therapy; adversarial methods should be carefully evaluated to ensure that they do not hallucinate anatomy or introduce HU bias that affects dose calculation.

For example, Chen et al^43^ trained a UNet model to generate sCT from CBCT, achieving a relatively good mean absolute error. Other UNet-based sCT generation from CBCT can be found in Liu et al^66^ and Thummerer et al.^67^ For ART, GAN-based models, especially CycleGAN was frequently used.^68^, ^69^, ^70^, ^71^ When these methods are intended for proton ART, dosimetric validation is essential. Some studies^71^, ^72^, ^73^ have evaluated sCT by recalculating the dose on the sCT and comparing the results with planning CT-based dose distributions, while others^43^, ^74^, ^75^ have focused primarily on image-quality metrics. Compared to the latter, by incorporating dosimetric-based equation, the former provides stronger and more comprehensive evidence for treatment-planning usability. Recent proton-therapy-specific studies^76^, ^77^, ^78^ have further emphasized the need to evaluate sCT clinical readiness using dose recalculation, normal tissue complication probability- or dose volume histogram (DVH)-driven endpoints, and adaptive decision consistency, particularly in head and neck (H&N) and nasopharyngeal cancer adaptive proton therapy.

There are also frameworks that utilized other DL-based models or combinations of UNet and GAN, for example, the SwinUNETR transformer architecture^79^ was applied to CBCT-to-sCT translation in prostate cancer, outperforming conventional UNet models in both image quality and dosimetric accuracy—an indication of the growing relevance of transformer-based DL in ART. Related work has also explored attention-guided CycleGAN, unsupervised DL-based sCT generation, and DL-enhanced 4-dimensional (4D) CBCT generation.^80^, ^81^, ^82^ Together, these studies show a broader shift from simple paired image translation toward methods designed to address practical limitations such as limited paired data, motion artifacts, respiratory phase inconsistency, and the need for anatomically faithful CBCT-derived images. For proton therapy, their clinical relevance depends on whether the generated images are validated for dose-calculation accuracy rather than image appearance alone.

Furthermore, several studies have explored the reconstruction of 3D CT images from MRI.^83^, ^84^, ^85^, ^86^, ^87^ These include studies using paired MR/CT learning, atlas- or registration-assisted approaches, and GAN-based or other DL-based models to infer CT-like HU information from MR images. MRI-to-sCT synthesis is attractive because MRI provides superior soft-tissue contrast without additional ionizing radiation, but its use for proton therapy remains limited because MRI does not directly provide electron density or RSP information. Therefore, MRI-derived sCT images require careful validation of HU/RSP accuracy and dose-calculation performance before they can be used for proton treatment planning. For example, MRI-only proton planning studies have evaluated whether GAN-generated sCT images can provide acceptable dosimetric accuracy for brain tumor proton therapy.^88^, ^89^ These studies support the feasibility of MRI-based sCT generation but also reinforce that clinical adoption requires site-specific geometric distortion management, HU/RSP validation, and proton dose-calculation verification.^51^, ^89^ At present, these approaches are not yet widely adopted in clinical proton therapy, partly due to hardware availability, workflow constraints, and remaining uncertainties in dose calculation.^90^ They are therefore only briefly discussed in this review.

### CT reconstruction from 2D inputs

Compared with reconstructing 3D CT from volumetric inputs such as CBCT, reconstructing CT from 2D images, although more clinically realistic, is considerably more challenging, as the information preserved in 2D projections is inherently sparse relative to full 3D CT data. Theoretically, this task is possible because 2D x-ray projections encode line-integral information through the patient, and multiple projections can constrain the underlying 3D attenuation distribution. However, when only 1 or 2 projections are available, the inverse problem is severely underdetermined: many different 3D anatomies can produce similar 2D projection images. AI-based methods attempt to address this by learning anatomical priors from training data and by combining these priors with projection geometry, ray-tracing consistency, multi-view information, or physics-informed constraints. The motivation for this direction is that orthogonal kV images are already widely acquired for patient setup, have relatively low imaging doses, and can be obtained quickly at the treatment position. If reliable 3D CT images could be reconstructed from such 2D images, they could potentially support improved patient alignment, anatomical change assessment, and adaptive decision-making without requiring repeated volumetric CT imaging. However, for proton therapy, such reCT images must be assessed not only for anatomical plausibility but also for their suitability for RSP estimation and dose calculation.

To validate feasibility, early studies investigated AI-driven reconstruction of 3D CT from digital reconstructed radiographs (DRRs),^91^, ^92^, ^93^, ^94^, ^95^, ^96^, ^97^ which are clean, noise-free 2D images generated from planning CT or CToR scans. These DRR-based studies are useful for testing whether neural networks can learn a mapping from projection-like images to volumetric anatomy under controlled conditions. While conceptually inspiring, such approaches are not clinically applicable, as DRRs themselves require prior 3D imaging for generation. From a practical standpoint, only independently acquired 2D images, such as kV projections, are qualified as meaningful input for 2D-to-3D reconstruction models. Although some studies^98^, ^99^, ^100^ explored x-ray-based training and testing, these images were often synthesized from 3D volumes using ray-tracing, thereby enforcing a strict one-to-one correspondence that did not reflect clinical reality. This difference is important because clinically acquired kV images contain scatter, noise, calibration differences, patient setup variation, and anatomical changes that may not be captured by synthetic projections.

Recently, however, a framework termed kV2CTConverter^49^ was introduced, which directly reconstructs 3D CT from clinically acquired kV images without reliance on prior CT. Results demonstrated both feasibility and near real-time performance, underscoring its use for more accurate 3D patient positioning and potential for ART image guidance. In that study, reconstruction performance was assessed using image-similarity and anatomical agreement measures between reCT and reference CT images, and the reCT showed the ability to capture patient-specific anatomical information inferred from the kV projections. Its potential value for ART is that a daily kV pair could be converted into an approximate 3D anatomical representation to support 3D setup assessment, detection of anatomical changes, adaptive triage, and selection of patients who require full volumetric imaging and dose recalculation. Nevertheless, as with other 2D-to-3D reconstruction methods, further evaluation is needed to determine whether the generated HU values and derived RSP maps are sufficiently accurate for proton dose recalculation, range verification, and adaptive replanning. Therefore, at present, such methods should be viewed as promising tools for anatomical assessment and ART decision support rather than as fully validated replacements for CT-based proton dose calculation.

Despite these advances, several challenges remain before 2D-to-3D CT reconstruction can be fully integrated into clinical proton therapy. A key limitation arises from the severe data sparsity of 2D projections, making the reconstruction problem inherently ill-posed and highly sensitive to noise, motion, and anatomical variability. Furthermore, the absence of strict correspondence between kV images and ground-truth CTs complicates supervised training, necessitating reliance on synthetic datasets or registration-based methods that may limit generalizability. Robustness against motion artifacts and anatomical changes is another open concern, particularly given the stringent dosimetric requirements of proton therapy. For example, a reCT may appear anatomically reasonable but still contain local HU errors, blurred tissue boundaries, missing soft-tissue changes, or incorrect air/tissue interfaces. These errors may be acceptable for patient-positioning assessment but unacceptable for proton dose calculation if they alter the water-equivalent path length along the beam direction. Therefore, future 2D-to-3D CT studies for proton therapy should include dosimetric validation, including HU/RSP error analysis, beam-path-specific range assessment, dose recalculation, and agreement of adaptive decisions with reference CT-based decisions. These validation requirements are more stringent than those for many photon setup-imaging applications, because in proton therapy a small local error near the beam path or distal edge can affect range and target coverage.

Future research directions include integrating physics-informed priors such as ray-tracing or MC–based projection operators to constrain the learning process, as well as leveraging multi-view acquisitions and data-efficient learning strategies. Additional directions include uncertainty-aware reconstruction, patient-specific fine-tuning, integration with prior planning CT information when clinically appropriate, and explicit modeling of projection geometry and scatter. Finally, large-scale, multi-institutional validation will be critical to establishing the clinical reliability of these models and to enabling their deployment in real-time adaptive proton therapy workflows. Such validation should assess not only image reconstruction accuracy but also proton-specific dose-calculation usability and adaptive decision consistency.

## Deformable image registration

During a treatment course, aligning the initial planning and subsequent verification images within a common coordinate framework is the foundation for subsequent image-based procedures. DIR is the process of spatially aligning two or more medical images by estimating a dense, voxel-wise deformation vector field (DVF) that maps anatomical structures from one image to another while accounting for complex, non-rigid changes such as organ motion, deformation, and anatomical variation. Therefore, DIR plays a critical role in ART workflows. Although the basic mathematical and image-processing requirements for DIR are similar between photon and proton therapy, the clinical consequences of DIR uncertainty can be more pronounced in proton therapy. This is because propagated anatomy, contours, and accumulated dose can influence patient-specific proton range and dose distributions, particularly when anatomical changes occur along the beam path.^101^, ^102^, ^103^ In photon therapy, registration errors may primarily affect contour propagation, dose accumulation, or local dose estimates; in proton therapy, they can additionally alter the tissue composition encountered by the beam and thereby affect range-sensitive dose distributions.^102^, ^103^ These effects may be particularly relevant in the presence of tissue interfaces, air cavities, bowel gas, sinus filling, or weight-loss-related anatomical changes. Therefore, in proton therapy, DIR should be evaluated not only by geometric image alignment but also by its downstream impact on dose accumulation, range evaluation, and adaptive decision-making.^101^, ^103^, ^104^ Here, we briefly summarize representative DL-based DIR approaches and their relevance to proton therapy; readers are referred to the review by Zou et al^105^ for a more comprehensive discussion of general DL-based medical image registration.

DL-based DIR approaches can generally be categorized into 3 classes according to the learning paradigm: unsupervised learning, supervised learning, and learning with joint tasks. Among these, unsupervised learning is the predominant paradigm for DL-based DIR. The unsupervised approach avoids the need for ground-truth DVFs^106^, ^107^, ^108^ by optimizing a loss function based on similarity metrics between the fixed image and the warped image generated from the moving image and the predicted DVF. The major advantage of this approach is that it can be trained using image pairs without requiring known ground-truth deformations, which are rarely available in clinical data. However, its performance depends strongly on the selected image-similarity metric and regularization strategy, and it may be challenged by intensity inconsistency, artifacts, large anatomical changes, or multimodal image pairs. In contrast, fully supervised approaches require the availability of ground-truth DVFs.^109^, ^110^, ^111^, ^112^ Supervised methods can directly learn from known deformation fields and may achieve accurate and fast inference when reliable labels are available, but such labels are difficult to obtain in real patient images and may depend on simulated or algorithm-generated DVFs. Joint learning frameworks leverage knowledge from additional tasks or modalities to achieve more comprehensive and accurate DVF predictions by incorporating contour, segmentation, uncertainty, or dose-related information. For proton therapy, all 3 learning paradigms require downstream validation because visually acceptable registration may still produce clinically relevant errors in propagated contours, accumulated dose, or range-sensitive anatomy.

### Unsupervised DIR approaches

VoxelMorph^106^ was among the first DL–based DIR methods and is widely regarded as a foundational baseline for subsequent approaches. It is an unsupervised framework that employs a UNet backbone combined with a Spatial Transformer Network (STN) to apply the predicted DVF to the moving image during inference, enabling rapid DVF prediction. Its strength lies in its simplicity, computational efficiency, and ability to train without ground-truth DVFs. However, because training is driven mainly by image similarity and deformation regularization, VoxelMorph-like methods may generate anatomically implausible deformations when image contrast is poor, artifacts are present, or large anatomical changes occur. This limitation is clinically important in proton therapy because a smooth or visually acceptable deformation may still incorrectly move tissue boundaries along a proton beam path, leading to inaccurate dose accumulation or range assessment.

Subsequent studies have enhanced VoxelMorph’s performance across various image modalities and anatomical sites by incorporating strategies such as random masking during training,^37^ probabilistic modeling^113^ of DVFs, cycle-consistency losses^114^ to stabilize training, and efficient hyperparameter tuning.^115^ These extensions attempt to improve robustness, estimate uncertainty, or reduce sensitivity to hyperparameter choices, but each modification addresses only part of the DIR problem, and none guarantees anatomically correct voxel-wise correspondence in regions with poor contrast, large deformation, or changing anatomy. Therefore, they do not completely remove the need for clinical plausibility checks and task-specific validation.

For large or complex cases, sequential or path-wise registration strategies have been proposed to improve accuracy, which is particularly critical for reliable dose accumulation in ART. Examples include multi-scale registration in LapIRN,^116^ longitudinal registration in Seq2Morph,^36^ and other related approaches.^117^, ^118^, ^119^, ^120^ Multi-scale and cascaded approaches can better handle large deformations by estimating motion progressively from coarse to fine scales, while longitudinal or path-wise approaches can exploit temporal relationships across serial images. These strategies may improve registration accuracy for adaptive workflows, but they can also increase model complexity and may accumulate errors across sequential steps if not carefully controlled. Additionally, vision transformer (ViT)-based models have been explored for DIR, achieving more global and generalizable DVFs.^107^ Compared with purely convolutional models, transformer-based methods may better capture long-range anatomical relationships, although they often require larger datasets and greater computational resources. Recent gradient-based DL-DIR frameworks, such as UniGrad^121^ and MultiGrad,^122^ further explore whether gradient information and multi-objective learning can improve registration generalizability. Their clinical value for adaptive proton therapy remains to be demonstrated using endpoints such as dose-accumulation accuracy, water-equivalent path length preservation, and agreement with replanning decisions.

### Supervised DIR approaches

Quicksilver^123^ is a patch-wise DL framework that predicts Large Deformation Diffeomorphic Metric Mapping momentum in a supervised manner, retaining diffeomorphic properties while achieving fast inference. Within the study, it is demonstrated that supervised DIR DL methods can approximate computationally expensive conventional registration while preserving desirable transformation properties. During training, Quicksilver learns from reference deformation information generated by a conventional registration framework and then predicts similar transformations much more rapidly during inference. The framework was further extended with a probabilistic formulation for estimating uncertainty in the predicted deformations and a correction network designed to improve registration accuracy.^123^ The advantage of supervised approaches is that they can directly learn deformation behavior from reference DVFs and provide very fast inference after training. Their main limitation is the requirement for high-quality training labels, which are difficult to define for clinical DIR because true voxel-wise anatomical correspondence is usually unknown. As a result, supervised models are often trained using synthetic deformations or deformation fields generated by conventional registration algorithms, which may transfer the limitations of those reference methods into the DL model.

Other approaches leverage weak or contour supervision to guide registration. For example, Hu et al^124^ used high-level anatomical labels during training but operated label-free at inference, enabling real-time multimodal (MRI–US) registration with high accuracy. This type of weakly supervised method is important because it reduces dependence on dense ground-truth DVFs while still incorporating anatomical information into model training. In radiotherapy, similar strategies are relevant when contours or anatomical labels are more available than true deformation fields. However, contour-guided supervision may improve alignment of labeled structures without guaranteeing accurate deformation in unlabeled tissues along proton beam paths. Therefore, for proton therapy applications, supervised or weakly supervised DIR should be evaluated not only by agreement with labels or contours but also by whether the deformation preserves density and anatomy relevant to stopping power and range.

### Learning with Joint tasks

Despite these advances, both unsupervised and supervised DL-DIR approaches have limitations.^101^, ^125^ Unsupervised methods, while flexible, can be sensitive to intensity variations, noise, or anatomical inconsistencies, potentially resulting in suboptimal or non-physical deformations. Supervised methods, on the other hand, rely on accurate voxel-level correspondences or high-quality ground-truth DVFs, which are often difficult or impossible to obtain for complex anatomies or multimodal images. These challenges have motivated the development of joint-task or multi-task DL-based DIR frameworks, which simultaneously optimize registration along with related tasks—such as segmentation, contour guidance, or dose accumulation—enhancing accuracy, robustness, and clinical applicability. For example, Liang et al^126^ proposed CT-to-CBCT DIR for H&N auto segmentation, Xie et al^127^ developed an unsupervised GAN-based STN for longitudinal CBCT abdominal RT, Smolders et al^128^ introduced DVF prediction with integrated plausibility scoring, and Hemon et al^129^ demonstrated contour-supervised DIR for prostate CBCT-guided RT. Together, these studies illustrate how auxiliary anatomical, temporal, plausibility, or contour information can make DL-DIR more clinically usable than models optimized only for image similarity.

However, the benefit of joint-task learning is not automatic. These frameworks often require additional annotations, such as contours, segmentations, uncertainty labels, or dose information, which may be expensive to obtain and may vary across institutions. They can also bias registration toward labeled structures while leaving deformation accuracy in unlabeled regions uncertain. This limitation is especially important in proton therapy, where unlabeled tissues along the beam path can still affect RSP and range. In addition, optimizing multiple tasks may increase model complexity and make it difficult to determine whether performance gains come from better registration or from the auxiliary task itself.

For proton therapy, joint-task DIR frameworks are especially attractive because geometric alignment alone may not be sufficient to determine clinical acceptability. A DVF that produces good image similarity may still be inadequate if it leads to incorrect contour propagation, inaccurate dose accumulation, or misleading assessment of proton range changes. Therefore, future DL-DIR studies in proton therapy should evaluate not only conventional geometric metrics, such as target registration error, Dice similarity coefficient, or Jacobian determinant analysis, but also downstream dosimetric endpoints, including accumulated dose differences, DVH changes, water-equivalent path length changes, and adaptive replanning decisions. Such proton-specific validation will be necessary before DL-based DIR can be safely integrated into online or real-time adaptive proton therapy workflows.

## Contouring and AI-based contour quality assessment

### Contouring

The segmentation of targets and OARs is a crucial component of radiation treatment planning. Currently the manual segmentation of these structures is still the standard of care, which is not only tedious and time-consuming but also inevitably prone to inter- and intra-observer variability. Due to the repetitive nature, particularly for normal structures with relatively consistent anatomical boundaries, segmentation tasks constitute ideal candidates for AI-based auto-segmentation. Readers are encouraged to refer to prior reviews for a broader discussion of AI-based auto-segmentation in radiotherapy^130^, ^131^ and the clinical implementation, evaluation, and challenges of auto-contouring tools.^130^, ^131^

Over the past years, AI-based auto-segmentation on common medical images, such as CT, MR, and PET, has rapidly evolved since the introduction of CNN, leading to extremely diversified research-orientated models and various commercial solutions. Compared to successful application of AI-based auto-segmentation for OARs, the development of AI tools for target delineation has progressed more slowly.^131^, ^132^ This is because target delineation is not always determined by image appearance alone. In addition to CT, MR, or PET findings, physicians may incorporate diagnostic reports, pathology, endoscopy, operative findings, disease spread patterns, treatment intent, clinical guidelines, and prior treatment information when defining gross tumor volume (GTV), clinical target volume (CTV), or planning target volume (PTV). Therefore, unlike many OAR segmentation tasks, target delineation often does not have a simple one-to-one mapping from image intensity to contour. The difficulty of exact voxel-wise differentiation of affected tissue from surrounding normal tissue further limits the reliability and generalizability of fully automated target segmentation.

By far, UNet is the most dominating architecture across all disease sites. The hierarchical down-sampling encoder and up-sampling decoder, together with skip connections at each scale forms the U-shape network and enables it to effectively capture low-level and high-level features, leading to accurate segmentation results. Because of this balance between localization and contextual feature extraction, UNet and its variants remain widely used for OAR segmentation and have also been explored for target segmentation. The introduction of ViT^133^ further enriches the pure CNN encoder network with the CNN-transformer hybrid network.^134^, ^135^, ^136^ ViT-based models divide images into patches and use self-attention mechanisms to capture long-range spatial relationships, which may be useful when segmentation depends on broader anatomical context rather than only local image texture. To further enhance the auto-segmentation performance, a hybrid network combines the traditional model-driven methods guided by explicit anatomical and context information and the data-driven AI-based methods.^137^, ^138^ Dynamic and interactive editing strategies have also been integrated into AI models via reinforcement learning (RL) for iterative contour refinement.^139^, ^140^ These interactive or human-in-the-loop approaches may be particularly important for target delineation, where physician review and clinical context remain essential.

Although contouring is required in both photon and proton therapy, the downstream consequences of contouring uncertainty can differ. In photon therapy, contour errors mainly affect target coverage, OAR dose constraints, and plan evaluation. In proton therapy, these effects remain important, but contour errors may also influence beam arrangement, robust optimization, range evaluation, and dose near distal edges or tissue interfaces. Errors in external body, air cavity, bowel, sinus, or other beam-path-related contours may alter the estimated water-equivalent path length and therefore affect range-sensitive dose calculation. This is particularly important in online adaptive proton therapy, where auto-segmented or propagated contours may be used under time pressure for dose recalculation, replanning, and treatment approval. Therefore, although AI-based auto-segmentation can reduce workload and improve consistency, its clinical use in proton therapy should be accompanied by careful physician review and, when appropriate, dosimetric evaluation of contour-related uncertainty. Future models that integrate multimodal images with clinical information may improve target delineation, but robust validation across institutions, disease sites, imaging protocols, and contouring guidelines will be necessary before fully automated target contouring can be routinely adopted.

### AI-based contour quality assessment

With the fast evolution in AI algorithms, auto-segmentation tools have demonstrated remarkable potential for fast and consistent contour delineation. Nonetheless, the accuracy of the delineation still needs to be evaluated and approved slice by slice before those contours are adopted for subsequent steps in radiotherapy planning to achieve optimal and safe treatment. Such evaluation process is named contour QA in this review, analogous to the well-recognized terminology machine QA and patient-specific QA (PSQA), for the ease of narrative. In current clinical practice, this contour QA process still relies heavily on physician and dosimetrist review, because geometric acceptability does not always guarantee clinical acceptability. Metrics to evaluate the performance of auto-segmentations generally fall into 4 categories: intensity and inter-image geometric metrics (such as Dice similarity coefficient and Hausdorff distance), recorded time savings, subjective scoring, and dosimetric metrics.^34^, ^141^ Geometric and intensity metrics are the most commonly used, but dosimetric metrics are particularly important in treatment planning because small contouring differences may or may not translate into clinically meaningful dose differences.

Auto-segmentation contour QA can be performed in two general settings: single-contour-set assessment and comparative assessment. In single-contour-set assessment, only one auto-segmentation result is available, and the model must judge contour quality using information such as image intensity, contour shape, anatomical plausibility, uncertainty maps, or relationships with neighboring structures.^142^, ^143^, ^144^, ^145^, ^146^, ^147^, ^148^ In comparative assessment, the contour under evaluation is compared with another independent contour set or reference segmentation.^149^, ^150^, ^151^, ^152^

Comparative assessment is more straightforward because geometric metrics can be calculated directly when a reliable reference contour is available. However, its performance depends on the quality of the reference contour; if the reference is imperfect or generated by a similar model, shared errors may be underestimated. Therefore, comparative assessment can support contour review, but it does not eliminate the need for clinical review or dosimetric evaluation.

In contrast, evaluation of a single auto-segmentation set is more challenging because no independent reference contour is available. Early approaches used hand-crafted features, including intra-contour shape descriptors, inter-structure relationships, and image-intensity features, followed by conventional machine learning models.^145^, ^147^ These methods provide interpretable features but may struggle to capture complex anatomical variation. More recent approaches have used AI models to learn contour-quality features directly from images, contours, probability maps, or uncertainty maps.^142^, ^143^, ^144^, ^146^, ^148^ For example, Men et al used a deep active learning strategy for lung auto-segmentation QA,^142^ while Chen et al incorporated probability and uncertainty maps with CT images for breast contour QA.^143^ Zhao et al used ResNet-152 as a feature extractor followed by a one-class support vector machine to identify potentially unacceptable contours.^144^ Rhee et al trained a CNN-based tool to detect contour errors for OARs in H&N patients,^148^ and Zarenia et al developed a SegResNet-based QA tool for auto-segmented abdominal contours.^146^ ResNet is a residual neural network that uses skip or residual connections to enable the training of very deep CNNs and is commonly used as a feature extractor in image-analysis tasks.^153^ SegResNet is a segmentation-oriented residual network that combines an encoder-decoder architecture with residual blocks, allowing it to learn multi-scale anatomical features for voxel-wise prediction and contour QA. This architectural design reflects the evolution from handcrafted feature-based contour assessment toward DL models that integrate image appearance, contour geometry, and uncertainty information.

For proton therapy, contour QA has additional significance because contour errors can affect not only target coverage and OAR dose constraints but also beam-path selection, robust optimization, range evaluation, and adaptive replanning decisions. Therefore, when auto-segmented contours are intended for proton treatment planning, assessment should ideally include both geometric evaluation and downstream dosimetric assessment. For example, contour errors may be evaluated by recalculating or re-optimizing plans using corrected and uncorrected contours, followed by comparison of DVH endpoints, robustness metrics, and clinically relevant dose constraints. Such dosimetric assessment can help distinguish contour deviations that are geometrically noticeable but clinically negligible from those that may alter plan quality or patient safety. This is especially relevant for adaptive proton therapy, where contour propagation and auto-contouring may directly influence whether adaptation is triggered and whether the adapted plan remains robust.

Despite progress in AI-based contour QA, several challenges remain. Many studies are disease-site specific, use retrospective datasets, or focus on OARs rather than target volumes. In addition, there is no universally accepted threshold for determining when an auto-segmented contour is clinically acceptable, because acceptability depends on the structure, disease site, treatment technique, physician preference, and dosimetric consequence. Future work should focus on standardized contour-assessment benchmarks, uncertainty-aware models, integration with clinical review workflows, and validation using clinically meaningful endpoints. For proton therapy, such validation should emphasize whether contour assessment helps prevent clinically meaningful errors in target coverage, OAR sparing, range evaluation, and adaptive replanning decisions.

## Dose prediction and dose denoising

Accurate dose calculation is the cornerstone of radiotherapy planning and evaluation. Traditionally, dose distributions are calculated using physics-based algorithms such as pencil beam convolution (PBC),^154^, ^155^, ^156^, ^157^ collapsed cone convolution (CCC), and MC simulations.^158^, ^159^, ^160^, ^161^ Among these, MC remains the gold standard, especially in proton therapy, where its ability to model particle interactions and heterogeneities provides unmatched accuracy. This is particularly important for proton therapy because proton dose distributions are highly sensitive to tissue density, stopping-power error, anatomical heterogeneity, and beam-path changes. Compared with photon therapy, small local density or stopping-power errors may shift the Bragg peak or distal dose falloff; therefore, AI-based proton dose tools must be evaluated using range-sensitive and robustness-related endpoints, not only nominal dose similarity.

However, the clinical implementation of MC faces major obstacles: computational cost, long runtimes, and storage demand. These barriers are particularly acute in ART, where frequent verification dose calculation and replanning are required to account for anatomical and physiological changes. Consequently, there is an urgent need for methods that are both fast and accurate, enabling real-time ART workflows while maintaining confidence in dose distributions. In this context, near-real-time AI support should be viewed as rapid dose estimation, uncertainty screening, or optimization guidance rather than replacement of clinical dose calculation or plan approval.

Recent years have seen rapid development of DL methods aimed at overcoming these limitations. Two important but distinct strategies have emerged: dose prediction and dose denoising. Dose prediction aims to estimate a clinically desirable or achievable dose distribution directly from patient images, contours, beam information, and/or planning objectives, thereby supporting treatment planning, plan-quality estimation, or optimization guidance. Dose denoising, in contrast, aims to accelerate physics-based dose calculation by taking a noisy dose distribution, such as a low-statistics MC dose, as input and recovering a high-quality dose distribution that approximates a high-statistics MC calculation. Thus, prediction primarily addresses patient-specific planning or optimization guidance, whereas denoising primarily addresses accelerated dose calculation by refining an already computed physics-based dose. A schematic comparison of these 2 concepts is provided in Figure 2.

**Figure 2:**
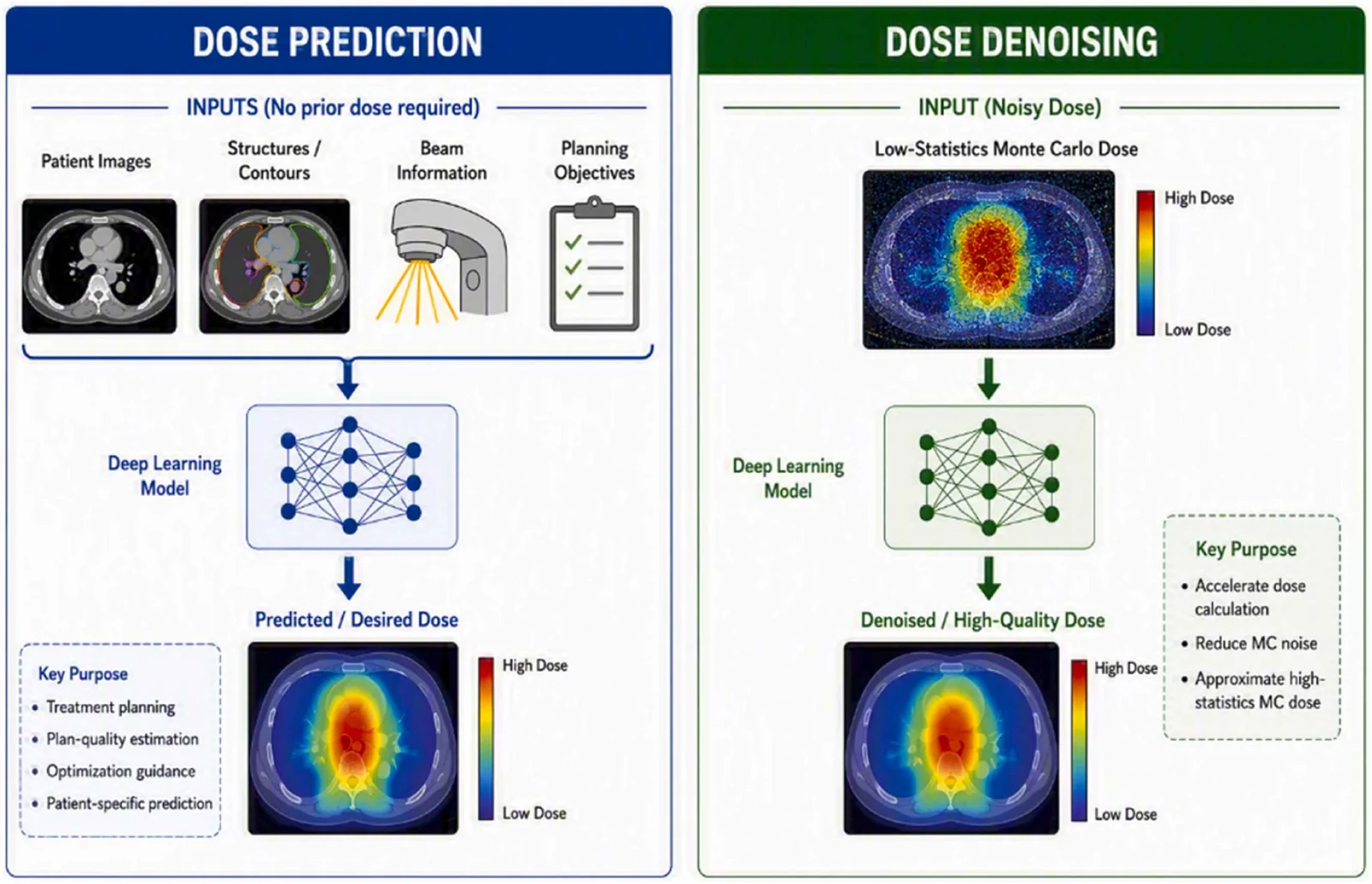
Schematic comparison of dose prediction and MC dose denoising.

### Dose prediction approaches

Dose prediction approaches learn a direct mapping from input images, such as CT, CBCT, sCT, or reCT, and/or structure contours, beam information, and planning objectives to dose distributions, bypassing some iterative planning steps and enabling rapid estimation of patient-specific dose distributions. Typical architectures include UNet variants^61^, ^162^, ^163^, ^164^ for voxel-wise regression, GANs^165^, ^166^ for sharper or more realistic dose maps, and more recently, transformer-based or hybrid models,^167^, ^168^, ^169^, ^170^ including ViT- and ResNet-based architectures, for long-range dependency modeling. These models can provide dose estimates within seconds after training, making them attractive for initial treatment planning, plan-quality estimation, adaptive decision-making, and rapid comparison of candidate plans.

Dose prediction is valuable in both photon and proton therapy, but its clinical role differs. In photon therapy, dose prediction has often been used to estimate achievable dose distributions for automated planning or plan-QA. In proton therapy, dose prediction may also support these tasks, but it must additionally account for beam range, energy-layer selection, spot placement, tissue heterogeneity, and robustness under setup and range uncertainties.^171^ Thus, proton dose prediction may be more challenging when models must generalize across beam arrangements, anatomical heterogeneity, and uncertainty scenarios. Conversely, when beam geometry and spot information are explicitly provided, proton models may benefit from the localized relationship between spot dose deposition and patient anatomy.

However, current DL-based dose-prediction models are typically disease-site or subsite specific. As a result, multiple models may be needed for different disease sites, prescriptions, beam arrangements, and institutional planning protocols, which is impractical for routine clinical use. In addition, many models are trained on relatively homogeneous datasets with standardized contours, prescriptions, and planning techniques. While such consistency facilitates model training, it may limit robustness when applied to patients with unusual anatomy, atypical targets, variable contouring practices, different beam configurations, or institutional planning differences. Future models should therefore improve generalizability, reduce dependence on strictly standardized inputs, and transfer across disease sites with minimal retraining or fine-tuning. Although one study^172^ proposed site-agnostic performance by training on a source site, such as prostate, and fine-tuning to another site, such as H&N, the evaluation remains limited.

Furthermore, in the absence of physics-based constraints, dose-prediction models may have limited physical interpretability. A predicted dose distribution may be visually plausible or statistically close to the training distribution but still violate beam-delivery constraints or inadequately represent heterogeneity-driven dose perturbations. This limitation is particularly relevant in proton therapy, where small errors in tissue stopping power, beam path, or anatomical change can cause clinically meaningful range and dose deviations. Zhang et al developed a physics-aware DL-based proton dose-prediction method using a noisy probing dose;^173^ however, such methods remain scarce. Recent proton-specific studies have also explored multi-site and variable-beam dose prediction,^174^ uncertainty estimation,^175^ and linear energy transfer (LET)-related dose-map prediction,^176^ reflecting growing interest in extending dose prediction beyond site-specific nominal-dose estimation.

Dose prediction can also serve as an intermediate step in automated treatment planning. Instead of replacing optimization, a predicted dose distribution may provide patient-specific objectives for inverse optimization or dose mimicking. This strategy has been studied in photon therapy and may be relevant to proton therapy, where predicted nominal or scenario-specific dose distributions could guide robust optimization. However, proton-specific implementation requires validation that the predicted dose remains compatible with deliverable spot weights, beam geometry, range uncertainty, and robustness requirements. Therefore, proton dose-prediction models should be evaluated not only by voxel-wise dose similarity but also by whether they can guide optimization toward clinically deliverable and robust plans.

In ART workflows, dose prediction may help triage whether full MC recalculation or replanning is needed, compare candidate adaptive plans, or provide initial objectives for inverse optimization. Scenario-specific dose prediction^60^, ^177^ may also estimate dose distributions under setup-shift or range-error scenarios before full scenario recalculation. In all cases, the predicted dose should be interpreted as a rapid decision-support or optimization-guidance tool rather than a stand-alone replacement for validated dose calculation. Confidence in the predicted dose must be established through comparison with physics-based dose calculations, DVH endpoints, robustness metrics, and clinical decision agreement.

### Dose denoising approaches

Dose denoising is distinct from dose prediction because its primary goal is to accelerate dose calculation by improving a noisy physics-based dose, typically a low-statistics MC dose. Due to the statistical nature of MC simulations, reducing noise requires a substantial increase in the number of simulated particles, which prolongs runtime. Therefore, high-fidelity MC simulation can be difficult to repeat frequently, especially in online adaptive workflows (see Section F) requiring repeated dose calculation in a short time (∼ minutes).

Dose denoising typically operates on an undersampled or low-statistics MC simulation. Conceptually, 3 dose-calculation options can be compared. First, high-statistics MC provides the most reliable physics-based dose estimate but requires longer computation time and may be difficult to use repeatedly in online ART. Second, low-statistics MC reduces runtime by simulating fewer particles, but it introduces stochastic noise that can affect voxel-wise dose, DVH metrics, optimization stability, and clinical interpretation. Third, DL-based denoising uses the low-statistics MC dose as input and learns to recover a dose distribution closer to a high-statistics MC reference. This strategy aims to retain much of the speed advantage of low-statistics MC while improving dose quality and clinical usability.^178^, ^179^

Unlike direct dose prediction, denoising starts from a physics-based dose calculation and learns to remove stochastic noise while preserving the underlying dose pattern determined by patient anatomy, beam arrangement, and particle transport. The model is usually trained using paired examples of low-statistics MC doses and corresponding high-statistics MC reference doses. During inference, the network receives a noisy MC dose and outputs a denoised estimate, ideally preserving clinically important features such as high-gradient regions, distal falloff, target coverage, and OAR dose. In proton therapy, dose denoising may be applied at different levels, including whole-plan dose^178^ and beamlet dose.^180^ Beamlet-level denoising can directly support optimization, whereas whole-plan forward-dose denoising primarily supports rapid plan evaluation or adaptive dose verification.

UNets^56^, ^181^ have been widely adopted to learn mappings between noisy and reference doses, demonstrating improved accuracy compared with conventional filtering. More advanced frameworks, such as GANs,^182^ have been applied to enhance structural preservation and reduce over-smoothing. GAN-based denoising may improve local dose texture and edge sharpness, although adversarial training can also introduce concerns regarding hallucinated features or reduced physical interpretability if not carefully validated. More recent studies are exploring transformer and diffusion models^183^ for improved generalization across patient cohorts and disease sites. These newer approaches may better capture long-range spatial dose relationships or complex noise-removal processes, but they remain at an early stage and require further validation for proton-specific dose accuracy and clinical robustness.

Compared with conventional post-processing filters, DL-based denoising can learn spatially and anatomically dependent noise-removal patterns rather than applying a fixed smoothing operation, which may help preserve dose gradients and structure-specific dose features. However, DL denoising can also introduce risks, including bias toward the training distribution, loss of rare but clinically relevant dose features, hallucinated structures, or underestimation of uncertainty. These risks are particularly important in proton therapy because inaccurate denoising near distal edges, heterogeneity interfaces, or beamlet-level dose contributions could affect range assessment or robust optimization.

Future directions for dose denoising include beamlet-level denoising, incorporation of MC kernels or dose-deposition models into DL networks, and uncertainty-aware frameworks that preserve physical consistency. For proton therapy, model evaluation should include not only voxel-wise dose error but also clinically relevant endpoints such as DVH differences, gamma analysis, target coverage, OAR dose metrics, range-sensitive regions, and robustness under setup and range uncertainty scenarios. Future studies should determine whether denoised low-statistics MC doses can reliably reproduce high-statistics MC results across disease sites, beam arrangements, anatomical changes, heterogeneous tissues, and individual beamlet or spot contributions. Multi-institutional validation will be needed before these models can be routinely used for clinical proton treatment planning or adaptive replanning.

## Optimization

Influence matrix (IM) is a central concept for conventional inverse treatment planning. By definition, an IM quantifies the dose contribution of each proton beamlet to each voxel within the selected patient volume of interest (VOI). By tuning the weight of each beamlet, the dose distribution in the VOI can be optimized to achieve uniform and conformal target coverage while keeping OAR doses as low as reasonably achievable. Robust optimization strategies, including worst-case^14^, ^17^, ^20^, ^184^ and probabilistic formulations,^21^, ^185^, ^186^ have been developed to safeguard against uncertainties in proton therapy by incorporating multiple uncertainty scenarios during optimization. These uncertainty scenarios commonly account for setup errors, range uncertainties, and, in some disease sites, motion-related anatomical variations. However, because one IM corresponds to one uncertainty scenario, IM-based robust optimization is computationally demanding due to the need to pre-calculate and store large numbers of IMs. The conventional trial-and-error iterative optimization process can be lengthy, sometimes requiring hours to converge.

Under traditional IM-based optimization schemes, AI can contribute in several complementary ways, including accelerating dose or IM calculation,^54^, ^180^ predicting achievable dose distributions,^162^ guiding inverse optimization,^187^ predicting spot weights directly, and supporting robustness evaluation across uncertainty scenarios.^60^, ^171^

Direct spot weight prediction has emerged as a promising strategy to accelerate treatment planning. By leveraging patient anatomy and desired dose distributions, DL models can directly predict spot weights, fluence maps, or other optimization variables, bypassing some computationally intensive steps in conventional inverse treatment planning. These predictive approaches can reduce the time required to generate plans of clinically acceptable quality to seconds or minutes, providing a natural bridge between accurate dose calculation, IM utilization, and efficient plan generation. This integration is particularly valuable in online ART, where rapid plan adaptation is required to accommodate anatomical changes, and near-real-time dose re-optimization becomes critical for maintaining treatment efficacy and safety. Several studies have contributed to treatment plan optimization through DL techniques,^188^, ^189^, ^190^, ^191^, ^192^ primarily in photon therapy, particularly intensity modulated radiation therapy (IMRT) and volumetric modulated arc therapy (VMAT). These works have demonstrated the feasibility of directly predicting fluence maps or optimization variables from patient anatomy and dose objectives.

Another important AI-assisted strategy is dose-prediction-guided optimization. In this approach, a DL model first predicts a patient-specific desirable dose distribution, which is then used as an input objective for inverse optimization or dose mimicking.^162^, ^193^ Dose mimicking aims to generate a deliverable treatment plan whose dose distribution closely reproduces the predicted dose while satisfying machine and clinical constraints. A typical workflow can be summarized in 3 steps (Figure 3). First, the prediction model estimates an achievable dose distribution from patient anatomy, contours, prescription, and optional beam information. Second, this predicted dose is translated into optimization objectives, voxel-wise reference doses, or structure-based constraints. Third, a physics-based optimizer or dose-mimicking algorithm searches for deliverable beamlet or spot weights that reproduce the predicted dose while satisfying machine, robustness, and clinical constraints. This workflow has been studied extensively in photon therapy, particularly for IMRT and VMAT, and provides a practical compromise between purely data-driven dose prediction and fully physics-based optimization. For example, prior dose-prediction and dose-mimicking studies have shown that predicted dose distributions can be used to guide automated plan generation, including cases with heterogeneous beam configurations.^194^

**Figure 3:**
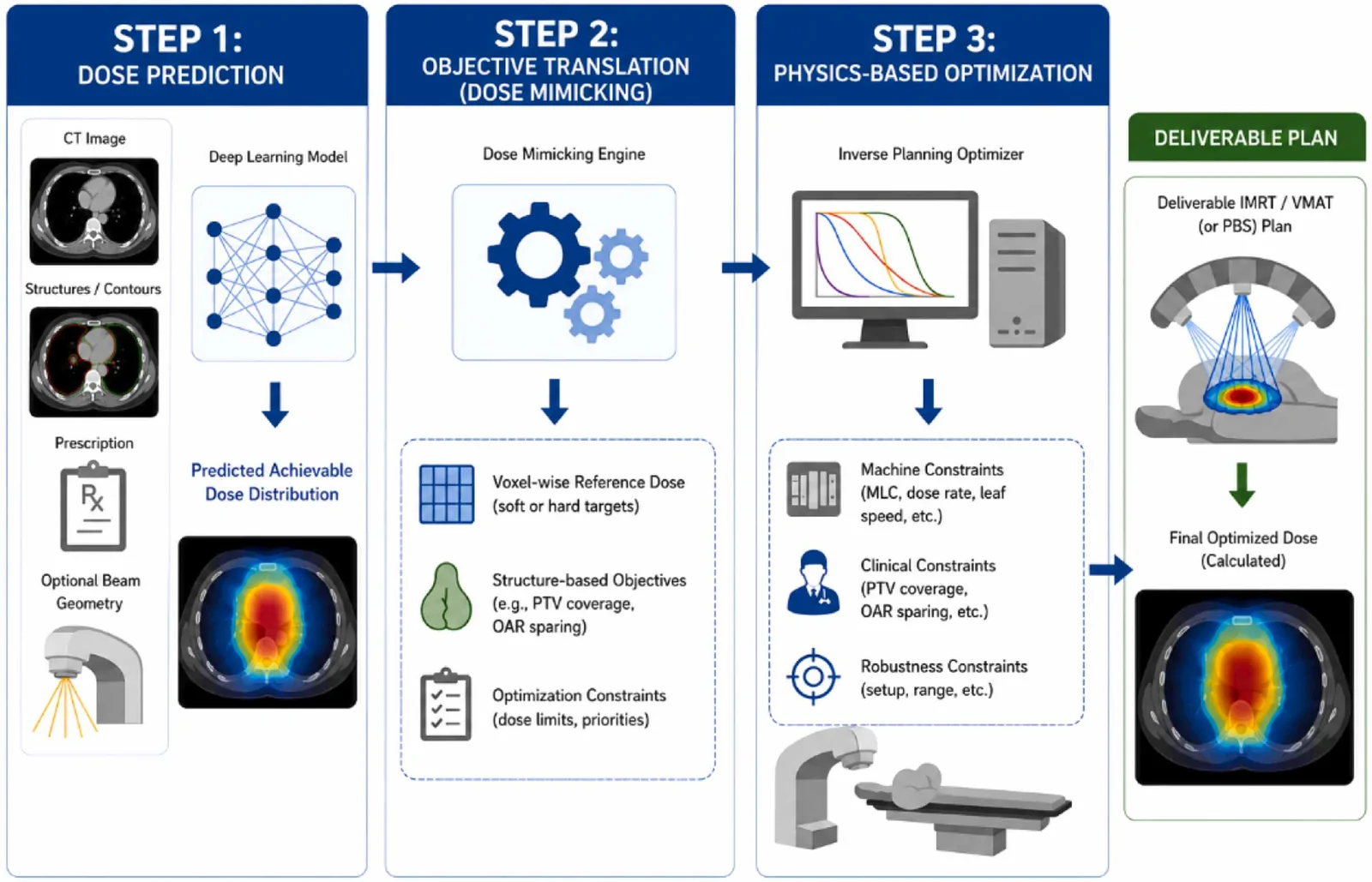
A typical workflow of DL-based dose prediction-guided dose mimicking.

However, because photon and proton dose distributions are governed by different physical mechanisms and delivery machine constraints, photon-based DL optimization models are not directly transferable to proton therapy without substantial modification and validation. Photon dose distributions are mainly governed by attenuation, scatter, beam modulation, and multi-leaf collimator-defined fluence, whereas proton dose distributions are strongly affected by finite range, tissue heterogeneity, and RSP uncertainties. Additionally, proton delivery machines specifically possess features like energy-layer sequencing, spot spacing, and machine delivery limitations. Therefore, translation of photon-based AI optimization methods to proton therapy requires proton-specific inputs and outputs, such as CT/RSP maps, beam geometry, spot maps, energy layers, and uncertainty scenarios. Validation should include deliverability and independent MC-based recalculation where possible. More importantly, due to the proton’s higher vulnerability to uncertainties, robustness is a core consideration for AI-based proton optimization. AI methods could extend the same concept of dose-prediction-guided optimization in photon by using predicted scenario-specific dose distributions^60^, ^177^ to guide robust optimization. For example, DL-based scenario-dose prediction for H&N intensity-modulated proton therapy (IMPT) has been investigated as a way to rapidly estimate robustness-related dose distributions and reduce the computational burden of scenario-based evaluation.^60^ Such approaches may substantially reduce the cost of robust optimization, but they also introduce questions regarding physical consistency, uncertainty propagation, and clinical acceptance criteria. If predicted scenario doses are biased or over-smoothed, the model may underestimate the worst-case target underdose or OAR overdose. Therefore, AI-based robustness tools should be validated using clinically relevant worst-case, probabilistic, or scenario-wise endpoints rather than only average dose error.

Several technical barriers remain before DL-based optimization can be fully integrated into routine clinical practice. These include limited interpretability of purely data-driven models, incorporation of uncertainty-scenario modeling for range and motion variations, and the need for large-scale, multi-institutional validation to establish generalizability across diverse patient cohorts and treatment protocols. AI-generated plans should be evaluated using both conventional plan-quality metrics and proton-specific endpoints, including target coverage, OAR sparing, robustness metrics, range-sensitive dose deviations, deliverability, and agreement with clinically accepted adaptive decisions. Additional barriers include the need for standardized benchmark datasets, consistent definitions of acceptable robustness, integration with commercial treatment planning systems, and prospective testing under realistic clinical time constraints.

Looking ahead, integration of DL with physics-informed constraints and MC-based dose-calculation engines represents a compelling direction for fast, accurate, and interpretable optimization frameworks capable of supporting real-time adaptive proton therapy. Rather than replacing inverse optimization entirely, AI may be most clinically useful within hybrid workflows, where learned models provide rapid predictions or guidance and physics-based optimization ensures deliverability, robustness, and clinical safety. In the near term, AI may reduce the search space, initialize optimization, prioritize candidate plans, or accelerate robustness screening, while final plan generation and approval remain grounded in physics-based dose calculation and expert clinical review.

## Adaptive radiation therapy

During the treatment course of proton therapy, inter-fractional anatomical changes may occur and lead to tumor underdosage or increased dose to surrounding OARs, potentially contributing to local recurrence or unexpected treatment-related adverse events. To address anatomical changes, ART has been introduced.^195^, ^196^ In ART, patients undergo periodic verification imaging during the treatment course to assess internal anatomical changes. Clinicians then evaluate whether the dose from the initial plan remains within clinically acceptable tolerance based on the verification images. If the initial plan no longer meets clinical requirements, adaptation or replanning may be triggered.

The implementation of ART involves nearly all key components of routine radiotherapy, including image acquisition, structure segmentation, dose verification, plan optimization, and PSQA. As a result, ART introduces substantial additional workload related to personnel effort, equipment uses, financial cost, and treatment-room time. As ART workflows evolve from delayed offline adaptation between fractions, to online adaptation before a fraction, and ultimately to real-time adaptation during a fraction, the demand for fast and reliable decision-making increases substantially. This challenge is particularly important in proton therapy because anatomical changes can alter water-equivalent path length and proton range and deteriorate plan robustness more severely than in photon therapy. Therefore, ART provides an important, although challenging, setting in which AI tools may offer substantial clinical value.

Many ART concepts and AI tools were first developed or implemented in photon therapy, including auto-segmentation, sCT/reCT generation, DIR-based contour propagation, dose prediction, adaptive triage, and automated replanning.^197^, ^198^ These tools provide important methodological foundations for proton ART because the overall workflow components are similar. However, direct transfer from photon ART to proton ART is not straightforward. In addition to target coverage and OAR sparing, proton ART must verify HU/RSP accuracy, water-equivalent path length, range uncertainty, scenario-wise robustness, and proton-specific deliverability. Therefore, photon-derived AI tools could be adapted for proton therapy, but they require proton-specific validation before clinical implementation.

AI tools developed for routine proton therapy planning can also support ART because the key workflow components substantially overlap. However, ART provides additional patient-specific information from previously delivered fractions, which may enable specialized models for anatomy monitoring, replanning decisions, and adaptive optimization. Rather than repeating the method details reviewed in previous sections, this section focuses on how these tools may be integrated into adaptive proton therapy workflows and what additional validation is required for ART-specific clinical use.

(1) Image acquisition and synthetic CT generation. As discussed in Section A, CT, CBCT, kV imaging, and MRI each offers different tradeoffs for adaptive workflows. CT provides reliable HU information for dose calculation but is less practical for frequent or online imaging, whereas CBCT and kV imaging are more accessible for on-board guidance but require correction or reconstruction before they can support proton dose calculation. For adaptive proton therapy, the key requirement is whether AI-corrected or reconstructed images can support HU/RSP accuracy, dose recalculation, range evaluation, and adaptive decision-making, rather than whether they only improve visual image quality.
(2) Image registration and contour propagation. As reviewed in Section B, AI-based DIR may accelerate contour propagation and dose accumulation by rapidly estimating the DVF between planning and verification images. In adaptive proton therapy, DIR errors may propagate into accumulated dose estimates, contour positions, water-equivalent path length assessment, and replanning decisions. Therefore, AI-based DIR for ART should be validated using not only geometric registration metrics but also downstream dosimetric and decision-based endpoints.
(3) Dose verification and adaptive decision-making. A central step in ART is determining whether the delivered or projected dose based on updated anatomy remains clinically acceptable. AI-based dose prediction and dose denoising may accelerate this process by rapidly estimating verification dose or approximating high-statistics MC dose calculation. In photon ART, fast dose prediction may help estimate whether target coverage or OAR constraints remain acceptable; in proton ART, the model must additionally account for range uncertainty, setup uncertainty, and scenario-specific dose changes. AI models may also help predict whether a patient is likely to require replanning based on early anatomical or dosimetric changes, thereby prioritizing clinical resources and reducing unnecessary adaptive procedures. However, such decision-support models should be evaluated by agreement with clinically accepted replanning decisions rather than by image or dose similarity alone.
(4) Plan adaptation and spot weight finetuning. Re-optimization is a central component of ART and can be performed either as full re-optimization with a new spot map or as spot-weight fine-tuning using the existing spot map or influence matrix. Full re-optimization may provide superior plan quality but is more complex and time-consuming, whereas spot-weight fine-tuning is simpler but may provide less improvement in adaptive plan quality. One study^199^ reported a model that could return spot weights based on the original influence matrix using the post-delivery verification dose. For proton therapy, any AI-assisted adaptive plan must satisfy deliverability, target coverage, OAR sparing, range uncertainty, and robustness requirements before clinical use. Therefore, adaptive plan evaluation should include not only nominal DVH metrics but also scenario-based robustness, machine deliverability, and consistency with clinical adaptation criteria.
(5) Anatomical and motion change prediction. For slow inter-fractional anatomical changes, AI can be used to predict anatomical evolution^200^, ^201^, ^202^, ^203^ so that adaptive considerations may be incorporated earlier, potentially during initial plan optimization or scheduled replanning. For fast intra-fractional anatomical changes, particularly in thoracic and abdominal regions, tumor motion can also be predicted using AI tools for real-time adaptation.^204^, ^205^, ^206^ These predictive approaches may help identify patients who are more likely to benefit from ART, optimize the timing of verification imaging, and support proactive replanning. Motion prediction is relevant in both photon and proton ART, but proton therapy may be more sensitive to motion-induced density and range changes, especially in thoracic and abdominal tumors where moving targets, lung density, bowel gas, or diaphragm motion can alter the water-equivalent beam path. Therefore, clinical use of such predictive models requires careful validation to avoid unnecessary adaptation or insufficient correction.

Overall, AI has the potential to support adaptive proton therapy by accelerating multiple steps, including image reconstruction, segmentation, registration, dose verification, re-optimization, PSQA, and anatomical change prediction. However, the clinical value of AI in ART should ultimately be measured by whether it improves the speed, consistency, robustness, and safety of adaptive decisions. Future studies should move beyond isolated task performance and evaluate complete AI-assisted ART workflows using clinically meaningful endpoints, multi-institutional validation, and prospective testing where feasible. This is especially important in proton therapy because errors from image generation, registration, contouring, dose calculation, and optimization can propagate into range assessment and adaptive replanning decisions.

## Large language models and multimodal AI in proton therapy treatment planning

LLMs represent an emerging class of foundation models with strong capabilities in language understanding, summarization, information extraction, reasoning, and structured report generation.^207^, ^208^ Radiation oncology is well suited for LLM integration because many workflows rely on textual and structured information, including clinical notes, pathology and radiology reports, prescriptions, simulation orders, plan-evaluation narratives, and dose-volume constraints. Although LLMs do not directly solve the physical challenges of proton therapy, such as HU/RSP conversion, range uncertainty, MC dose calculation, or robust optimization, they may provide workflow-level support by integrating clinical context, standardizing documentation, assisting plan review, and improving communication among clinicians, physicists, dosimetrists, therapists, and patients.^209^, ^210^, ^211^, ^212^, ^213^, ^214^, ^215^, ^216^, ^217^, ^218^, ^219^, ^220^

A key technical strategy for clinical LLM deployment is grounding. General-purpose LLMs may generate plausible but incorrect responses when source-of-truth information is unavailable.^207^, ^221^ Therefore, modern clinical LLM systems increasingly use retrieval-augmented generation (RAG),^222^ tool-augmented agents,^223^, ^224^ supervised fine-tuning,^214^ and modular designs that separate deterministic calculations from natural-language generation.^223^ In radiation oncology, this is particularly relevant because many tasks can be anchored to structured information, such as dose-volume statistics, prescription data, structure nomenclature, protocol constraints, and electronic health record-derived clinical variables. For proton therapy, such grounding is essential because LLM-generated outputs should not independently determine dose calculation, plan approval, or adaptive replanning decisions; instead, they should support auditable decision-making by retrieving verified information, summarizing constraints, and communicating results in a clinically interpretable format.

Several LLM applications are directly relevant to proton therapy planning workflows (Figure 4). First, LLMs can support clinical data integration by extracting diagnosis, staging, prior treatment, modality, fractionation, and treatment-intent information from unstructured notes.^225^, ^226^ Fine-tuned open-source LLMs have been investigated for radiation oncology tasks such as treatment regimen generation, modality selection, and international classification of diseases 10th revision (ICD-10) prediction, demonstrating that domain-specific curation and supervised fine-tuning can improve performance compared with general models.^214^ In proton therapy, similar approaches may help identify planning-relevant clinical factors, such as re-irradiation history, prior dose, surgical status, implanted devices, or clinical indications for proton therapy.

**Figure 4:**
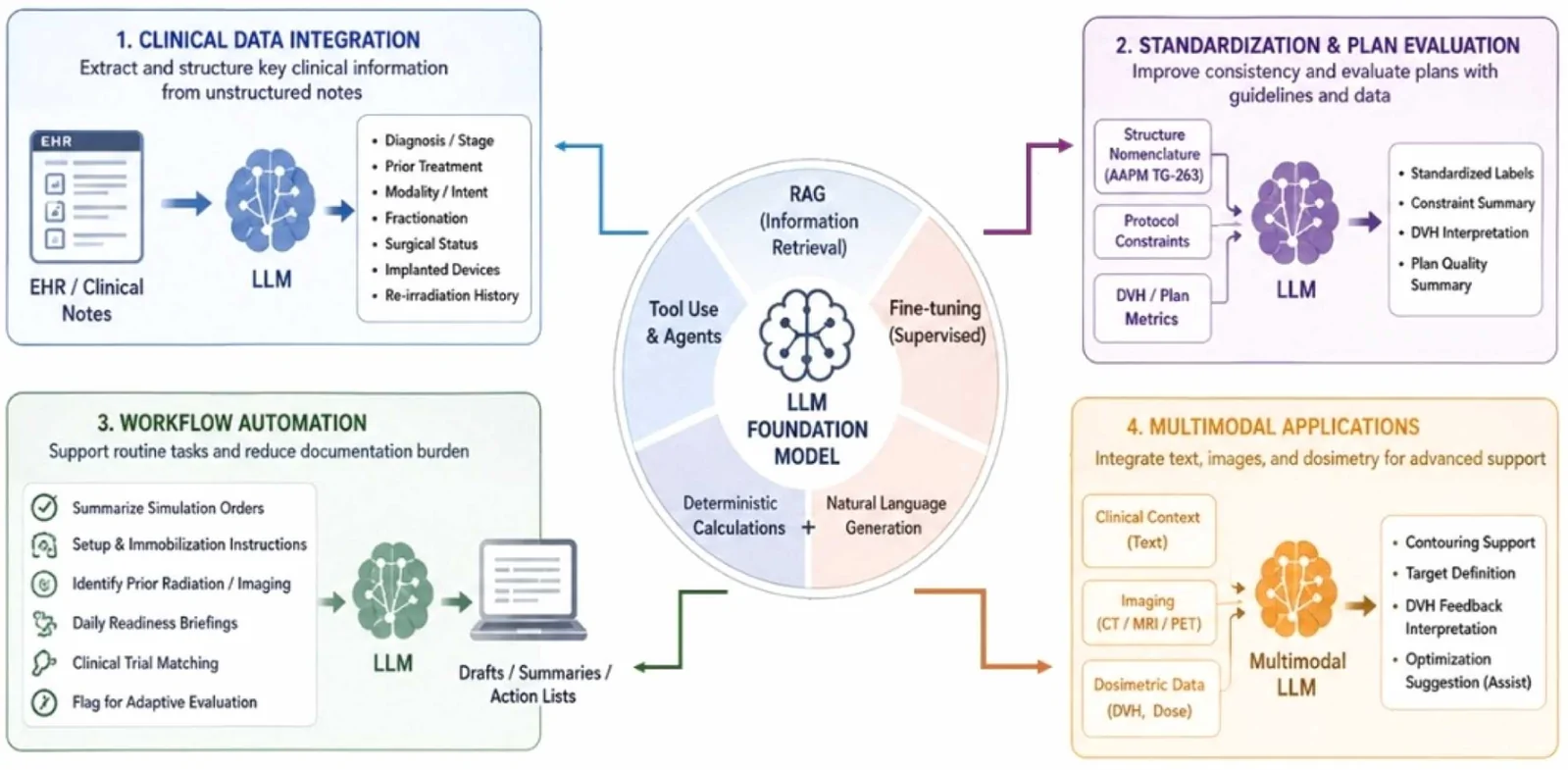
Schematic overview of technologies and tools used for deployment of LLM and the corresponding applications in proton therapy.

Second, LLMs may improve standardization and plan evaluation. Structure nomenclature standardization is an important prerequisite for automated contouring, dose accumulation, multi-institutional research, and treatment-plan evaluation. LLM-based systems have been evaluated for relabeling radiotherapy structures according to AAPM TG-263,^227^ illustrating how guideline language can be translated into structured outputs. For proton therapy, standardized structure names and consistent clinical documentation are particularly valuable because downstream analyses may depend on accurate matching of targets, OARs, beam-path structures, and dose-volume endpoints. LLMs may also support protocol-aware plan evaluation by combining RAG, structured DVH data, and deterministic constraint-checking modules. In this setting, the LLM should summarize independently computed plan-quality metrics rather than generate dose values or autonomous clinical judgments.

Third, LLMs may assist routine workflow automation. Electronic health record-integrated systems have been used to draft patient-message responses, summarize CT simulation orders, generate daily readiness briefings, and support clinical trial matching.^228^, ^229^, ^230^ For proton therapy planning, the most relevant applications include summarizing simulation orders, standardizing setup and immobilization instructions, identifying prior radiation or pending imaging, and flagging patients who may require adaptive evaluation.^208^ These tools may reduce documentation burden and improve workflow consistency, particularly for complex patients requiring multidisciplinary coordination.

Fourth, emerging multimodal LLM systems may support contouring and treatment planning by integrating clinical text with imaging and dosimetric data.^231^, ^232^, ^233^, ^234^ Target delineation often depends on clinical context beyond image appearance alone, including tumor stage, laterality, surgical history, pathology, prior treatment, and physician intent. LLM-augmented multimodal segmentation frameworks may therefore improve target definition by incorporating patient-specific clinical information into image-based segmentation models.^231^, ^232^ Similarly, early studies have explored whether LLMs can assist treatment planning by interpreting DVH feedback and suggesting optimization parameter adjustments.^233^, ^234^ For proton therapy, such applications remain preliminary and require additional validation because any planning recommendation must remain compatible with proton beam geometry, spot delivery, range uncertainty, robustness requirements, and institutional planning practice.

Finally, safe clinical deployment of LLMs requires careful attention to failure modes, validation, privacy, and governance.^221^, ^235^, ^236^, ^237^ Common risks include hallucination, omission of key clinical details, inappropriate generalization, prompt sensitivity, overconfident wording, and vulnerability to untrusted inputs. These risks are especially important in proton therapy because incomplete or inaccurate clinical context could affect planning decisions, adaptive triage, or interpretation of prior dose and anatomical change. Therefore, LLM outputs should generally be treated as drafts, summaries, or decision-support information rather than autonomous clinical decisions. Practical safeguards include RAG-based grounding, source citation, deterministic constraint-checking, audit logs, secure deployment for protected health information, and human-in-the-loop review by clinicians and physicists.^223^

Overall, LLMs and multimodal foundation models should be viewed as workflow-support and clinical-intelligence tools that complement, rather than replace, physics-based AI models for proton therapy. Their near-term value lies in documentation, standardization, information retrieval, plan-review communication, adaptive workflow coordination, and patient engagement. Prospective validation, failure-mode analysis, privacy safeguards, and workflow-level evaluation will be necessary before routine clinical integration.

## Discussion

AI applications in proton therapy span multiple domains, including imaging, dose calculation, plan optimization, adaptive therapy, and treatment-planning QA. Each area shows encouraging progress, yet several overarching challenges remain. First, many DL models lack physical interpretability, which limits clinical trust and hinders safe deployment. Incorporating physics-informed constraints, such as dose kernels or MC approximations, can improve both robustness and transparency. This is particularly important for proton therapy, where visually plausible AI-generated images or dose distributions may still be clinically inadequate if they do not preserve RSP accuracy, proton range, or scenario-specific dose behavior. Therefore, future studies should report both conventional performance metrics and proton-specific dosimetric endpoints, including range error, DVH differences, gamma passing rates, and robustness metrics under setup and range uncertainty scenarios. Compared with photon therapy, these validation requirements are more stringent because proton dose distributions can be strongly affected by small errors in stopping power, anatomical heterogeneity, and beam-path anatomy.^238^

Second, uncertainty management remains particularly critical in proton therapy, where range uncertainty and anatomical change can substantially affect dose distributions. AI methods have been proposed for robustness evaluation,^59^, ^171^ scenario-specific dose prediction,^60^, ^177^ and robust dose mimicking,^162^, ^177^, ^193^ suggesting that AI may help identify plans vulnerable to uncertainty and accelerate robust plan adaptation. However, broader integration of AI-driven prediction with clinically validated robust optimization remains an active research area. Key unresolved questions include how to enforce physical consistency across uncertainty scenarios, propagate model uncertainty into robustness evaluation, and define acceptance criteria for AI-generated or AI-assisted robust plans. For adaptive proton therapy, these questions become even more important because image-generation errors, DIR uncertainty, contour uncertainty, and dose-calculation uncertainty may accumulate across the workflow and influence whether adaptation is triggered. Thus, AI-assisted proton therapy should be evaluated at the workflow level, not only at the level of isolated model outputs.

Third, the field suffers from limited availability of large, annotated datasets, restricting model generalizability across institutions and patient populations. This limitation is especially relevant for proton therapy because model performance can be affected by disease site, beam arrangement, delivery system, scanner type, reconstruction protocol, contouring practice, dose-calculation engine, and institutional planning philosophy. Many current studies remain retrospective, single-institutional, and disease-site specific.^64^ As a result, external validation, multi-institutional benchmarking, and transparent reporting of training data, validation cohorts, and clinical endpoints are essential before these tools can be adopted routinely. Studies should also clearly distinguish whether a model was developed specifically for proton therapy or adapted from photon/general radiotherapy applications, because adapted methods may require additional validation for RSP accuracy, range uncertainty, spot delivery, and robust optimization.

A recurring theme across this review is that many AI methods are shared between photon and proton therapy, but their clinical validation requirements differ. Image segmentation, DIR, dose prediction, sCT/reCT generation, and automated planning are important in both modalities. However, in photon therapy, errors often primarily affect local dose estimation, contour-based evaluation, or OAR/target dose metrics. In proton therapy, the same errors can also propagate into water-equivalent path length, beam range, distal-edge placement, scenario-specific dose, and robust optimization. Therefore, photon-derived AI tools provide valuable methodological foundations, but direct transfer to proton therapy usually requires additional inputs, such as HU/RSP maps, beam geometry, spot or energy-layer information, uncertainty scenarios, and proton-specific endpoints. This distinction should be considered when interpreting studies that are not proton-therapy-specific but are cited for methodological relevance.

Beyond conventional IMPT planning, AI may also contribute to emerging proton-specific applications, including proton arc therapy,^239^, ^240^ FLASH proton therapy,^241^, ^242^ LET-guided planning,^243^ range verification,^244^, ^245^, ^246^ and motion-adaptive delivery.^247^, ^248^ In proton arc therapy, AI may help navigate the enlarged optimization space created by many beam angles, energy layers, and spot trajectories. In FLASH proton therapy, AI may support rapid plan generation and evaluation under additional constraints related to dose rate, delivery time, normal-tissue sparing, and machine capability. AI may also support LET prediction, LET-guided optimization, motion-adaptive delivery, and range-verification methods such as proton-acoustic imaging.^246^ These applications remain early-stage but represent important proton-specific research directions in which AI may provide value beyond general radiotherapy automation.

As discussed in Section G, LLMs and multimodal AI may support workflow-level tasks, such as clinical-context extraction, protocol retrieval, planning documentation, plan-review assistance, adaptive workflow triage, and integration of textual clinical records with imaging and dosimetric information. However, these models must be distinguished from safety-critical models that directly affect dose calculation, optimization, or plan approval. Before clinical use in proton therapy decision support, LLMs and multimodal AI require rigorous validation, privacy protection, hallucination control, uncertainty quantification, failure-mode analysis, and integration into physician and physicist review workflows.

Another important consideration is the relationship between AI-based workflow acceleration and clinical responsibility. AI tools may reduce the time required for image reconstruction, DIR, auto-segmentation, dose calculation, plan optimization, and adaptive replanning, but faster workflows do not remove the need for independent verification.^249^ AI-generated sCT/reCT images, DIR results, contours, predicted doses, and spot weights should be reviewed against anatomical fidelity, dose-calculation usability, clinical objectives, robustness requirements, and deliverability. Thus, AI should currently be viewed as a decision-support and workflow-acceleration tool rather than a replacement for expert clinical judgment. This human-in-the-loop model is especially important in proton therapy because small model errors may have amplified downstream effects on range, robustness, and adaptive decisions.

Finally, while early studies demonstrate technical feasibility, few have undergone prospective validation, and regulatory pathways for AI adoption in radiation oncology are still evolving. Future directions should prioritize hybrid physics-AI frameworks, multimodal data integration, standardized evaluation benchmarks, and multi-institutional collaborations to ensure reproducibility, clinical reliability, and safe deployment. For proton therapy specifically, future research should emphasize proton-relevant endpoints, including RSP accuracy, range uncertainty, scenario-wise robustness, LET-related considerations where applicable, and the dosimetric impact of AI-generated inputs. Establishing such evaluation standards will be essential for moving AI-assisted proton therapy treatment planning from technical feasibility toward clinically reliable implementation. Ultimately, successful clinical translation will require not only accurate AI models but also workflow-level validation showing that AI improves planning efficiency, adaptive decision consistency, robustness, and patient safety.

## Conclusion

AI holds strong potential to transform proton therapy by enabling faster, more consistent, and more adaptive treatment planning and delivery. Across the proton therapy planning chain, AI methods may support imaging, registration, segmentation, contour assessment, dose prediction and denoising, optimization, robustness evaluation, and adaptive replanning. LLMs and multimodal foundation models may further support documentation, clinical-context integration, and workflow coordination, but they should remain distinct from safety-critical dose calculation or plan-approval tasks. Widespread clinical adoption will require interpretable, physics-aware models; validation in large and diverse patient cohorts; and evaluation using proton-specific endpoints such as HU/RSP accuracy, range uncertainty, dose-calculation accuracy, robustness, deliverability, and adaptive decision consistency. With continued interdisciplinary collaboration and workflow-level validation, AI-assisted proton therapy can advance toward safer, more efficient, and more personalized cancer care.

## Funding

This research was supported by NIH/BIBIB R01EB293388, by NIH/NCI R01CA280134, by the Eric & Wendy Schmidt Fund for AI Research & Innovation, and by the Kemper Marley Foundation.

## CRediT authorship contribution statement

Y.D. and H.F. were responsible for data curation, formal analysis, methodology development, and writing of the original draft. W.L. contributed to conceptualization, project administration, supervision, and writing and editing. All authors participated in technical discussions, manuscript review, and editing.

## Declaration of Conflicts of Interest

The authors declare the following financial interests/personal relationships, which may be considered as potential competing interests: Wei Liu reports a relationship with the National Institutes of Health that includes: funding grants. Wei Liu reports a relationship with The Eric & Wendy Schmidt Fund for AI Research & Innovation that includes: funding grants. Wei Liu reports a relationship with The Kemper & Ethel Marley Foundation that includes: funding grants. Wei Liu has patent issued to System and Method for Robust Intensity-Modulated Proton Therapy Planning. Wei Liu has patent issued to System and Method for Novel Chance-Constrained Optimization in Intensity-Modulated Proton Therapy Planning to Account for Range and Patient Setup Uncertainties. Wei Liu: Participation on a Data Safety Monitoring Board or Advisory Board: Red Journal, Medical Physics, Green Journal, IEEE TMI, PMB, JACMP, NIH Image Technology Development Study Section, Special Emphasis Panel (ZRG1 SBIB-Q 03) of NIH. If there are other authors, they declare that they have no known competing financial interests or personal relationships that could have appeared to influence the work reported in this paper.

## Data Availability

Research data are stored in an institutional repository and will be shared upon request to the corresponding author.
